# Real-World Treatment Patterns and Healthcare Resource Utilization in Tardive Dyskinesia: Claims Analysis of Individuals Treated with Benztropine or a VMAT2 Inhibitor

**DOI:** 10.36469/001c.169275

**Published:** 2026-11-13

**Authors:** Craig Chepke, Handing Xie, Sagnik Chatterjee, Pooja Gandhi, Amy Yang

**Affiliations:** 1 Excel Psychiatric Associates, Huntersville, NC, USA; 2 Atrium Health, Charlotte, North Carolina, USA; 3 Teva Branded Pharmaceutical Products R&D LLC, West Chester, Pennsylvania, USA; 4 KMK Consulting, Inc., Morris Plains, New Jersey, USA; 5 Teva Branded Pharmaceutical Products R&D LLC, Parsippany, New Jersey, USA

**Keywords:** tardive dyskinesia, movement disorders, VMAT2 inhibitor, benztropine, anticholinergics, healthcare resource utilization

## Abstract

**Background:**

Tardive dyskinesia (TD) is a persistent hyperkinetic movement disorder associated with the use of dopamine receptor antagonists. Despite vesicular monoamine transporter type 2 (VMAT2) inhibitors being recommended as first-line treatment, benztropine is sometimes prescribed off-label and may aggravate TD symptoms.

**Objective:**

To compare US healthcare resource utilization (HCRU) between benztropine-treated and nontreated and between benztropine-treated and VMAT2 inhibitor–treated individuals with TD.

**Methods:**

Retrospective claims analyses included individuals with TD with ≥1 year of continuous insurance coverage before and after treatment initiation (index date). Part 1 (MarketScan® database [July 2012–Sept. 2021]) included adults with first TD diagnosis from July 2013 to September 2020. Individuals with benztropine initiated on/after first TD diagnosis were matched exactly by age, sex, and the Quan adaptation of the Charlson Comorbidity Index to nontreated individuals. Part 2 (MarketScan® [Jan. 2016–Sept. 2023]; Medicaid [Jan. 2018–Dec. 2022]) included adults with TD diagnosis (Jan. 2017–Sept. 2022) ≤1 year before benztropine or VMAT2 inhibitor initiation. VMAT2 inhibitor–treated and benztropine-treated cohorts underwent 1:1 propensity score matching. All-cause HCRU was compared between matched cohorts.

**Results:**

In part 1, 92 benztropine-treated individuals with TD were matched to 92 nontreated individuals with TD (mean age, 47.7 years; 77.2% female). Post-index, mean (SD) annual number of emergency department visits was significantly higher for benztropine-treated individuals than nontreated individuals (1.0 [1.8] vs 0.5 [1.0]; *P* = .0232). In part 2, post-index, significantly higher proportions of individuals in the benztropine-treated cohort (n = 272) vs the matched VMAT2 inhibitor–treated cohort (n = 272) had ≥1 inpatient visit (36.8% vs 22.1%; *P* < .001). Mean (SD) numbers of inpatient (0.9 [2.2] vs 0.5 [1.2]; *P* = .004) and emergency department visits (2.6 [6.0] vs 1.6 [4.0]; *P* = .024) were higher in the benztropine-treated vs VMAT2 inhibitor–treated cohorts, respectively.

**Conclusion:**

Treatment of TD with benztropine was associated with higher all-cause HCRU than no treatment or VMAT2 inhibitor treatment.

## INTRODUCTION

Drug-induced movement disorders (DIMDs) associated with the use of dopamine receptor antagonists include tardive dyskinesia (TD), parkinsonism, akathisia, and dystonia.^1,2^ These disorders present with involuntary movements, and despite disparate phenomenology and treatment strategies, have often been lumped into the single term “extrapyramidal symptoms” (EPS).^3,4^ DIMDs can affect daily functioning and healthcare resource utilization (HCRU) and may impact adherence to antipsychotic medications.^5–8^ Treatment for DIMDs varies by movement disorder type and can include anticholinergics, antipsychotics, benzodiazepines, and vesicular monoamine transporter type 2 (VMAT2) inhibitors.^2,4^ Though the term EPS is nonspecific and may reflect one or more disorders that differ in pathophysiology and treatment, real-world studies have shown that many clinicians treat DIMDs uniformly, contrary to clinical evidence and guidelines.^9,10^

TD can be differentiated from other DIMDs by timing and presentation.^11,12^ TD is a hyperkinetic movement disorder characterized by involuntary, repetitive, and purposeless movements and is often irreversible.^13^ TD may begin after months to years of treatment with dopamine-receptor blocking agents and most commonly manifests with involuntary movements of the face, neck, trunk, and extremities.^14,15^ Despite common historical belief that they were not bothersome, mounting evidence suggests that the extra movements associated with TD may impact daily functioning and quality of life.^16^

Benztropine, an antimuscarinic, anticholinergic medication, is available in oral, intravenous, and intramuscular formulations and is indicated as an adjunctive therapy for various forms of parkinsonism.^17^ There is a lack of evidence supporting the efficacy of anticholinergics for TD.^18^ Specifically, benztropine prescribing information in the United States (US) and Canada contains a warning/precaution that benztropine may aggravate TD symptoms, and product data sheets from Australia/New Zealand list benztropine as contraindicated for TD.^19–22^ Despite this, benztropine is often prescribed off-label to individuals with TD in an attempt to provide symptom relief.^9^

Aside from the risk of potentially exacerbating the symptoms of TD,^23^ benztropine and other anticholinergics are associated with adverse events across organ systems.^17,24^ In older patients, anticholinergic burden has been associated with multiple negative outcomes. For example, in older adults without cognitive impairment, use of anticholinergic medication has been associated with increased brain atrophy and cognitive decline.^25^ Anticholinergic burden has also been associated with increased fall risk^26^ and an increased risk of developing dementia.^27,28^ For these additional reasons, it has been suggested to avoid anticholinergic use in elderly individuals who develop drug-induced parkinsonism^11^ and to avoid benztropine use in those with TD.^29^ Consistent with these recommendations, anticholinergic burden is known to exacerbate cognitive deficits associated with psychotic disorders,^30–32^ and reducing anticholinergic burden in individuals with schizophrenia or bipolar disorder has been associated with improvements in cognition and in quality of life.^33^

In 2017, two VMAT2 inhibitor drugs, deutetrabenazine and valbenazine, were approved by the US Food and Drug Administration (FDA) to treat TD.^34,35^ In phase 3 clinical trials, these VMAT2 inhibitors were shown to have significantly greater efficacy than placebo in reducing the abnormal involuntary movements associated with TD.^36,37^ Deutetrabenazine (once daily or twice daily) and valbenazine (once daily) are available in oral formulations. In the treatment of TD, the most common adverse events reported with deutetrabenazine were nasopharyngitis and insomnia, and the most commonly reported adverse event with valbenazine is somnolence.^34,35^

There is a scarcity of published data comparing outcomes of patients with TD prescribed benztropine to those of untreated patients or to those who are prescribed VMAT2 inhibitor drugs. This 2-part, real-world retrospective study used claims analysis to compare HCRU in individuals with TD. In part 1, HCRU was compared for individuals with TD treated with benztropine vs those who received no treatment. In part 2, clinical and demographic characteristics, treatment patterns, and all-cause HCRU were compared between individuals with TD who were prescribed benztropine and those who were prescribed a VMAT2 inhibitor.

## METHODS

### Study Design

**Part 1: Benztropine vs no treatment**: Claims data captured in the US-based MarketScan® research database, including commercial and Medicare claims, between July 2012 and September 2021 (study period) (**Supplementary Figure S1**) were used. Eligible individuals were aged ≥18 years and first diagnosed with TD (ie, *International Classification of Diseases, Tenth Revision* [ICD-10] code G24.01) between July 2013 and September 2020 (identification period). Inclusion criteria for the benztropine-treated cohort were a first benztropine prescription date (ie, index treatment date) on or after the first TD diagnosis date and ≥1 year of continuous insurance coverage from ≥1 year prior to and ≥1 year after the index treatment date. Individuals were eligible for the nontreated cohort if they had no claim for benztropine, valbenazine, or deutetrabenazine during the entire study period. Exclusion criteria for either cohort were any claim for a VMAT2 inhibitor during the entire study period, or diagnoses of Parkinson’s disease, other drug-induced secondary parkinsonism, neuroleptic-induced parkinsonism, Huntington disease, or EPS during the entire study period.

**Part 2: Benztropine vs VMAT2 inhibitor:** Claims data captured in the MarketScan® research databases, including commercial and Medicare claims (Jan. 2016–Sept. 2023), and in the multi-state Medicaid database (Jan. 2018–Dec. 2022) (**Supplementary Figure S2**) were used. Eligible individuals were diagnosed with TD between January 2017 (the year of VMAT2 inhibitor FDA approvals) and September 2022. Inclusion criteria were ≥1 pharmacy claim for benztropine or ≥1 pharmacy claim for a VMAT2 inhibitor, a TD diagnosis during the 12 months prior to or on the date of either a benztropine or a VMAT2 inhibitor pharmacy claim (index treatment date), aged ≥18 years on the index date, and continuous insurance coverage from ≥1 year prior to and ≥1 year after the index treatment date. Exclusion criteria were diagnosis during the entire study period with Parkinson’s disease, other drug-induced secondary parkinsonism, neuroleptic-induced parkinsonism, Huntington disease, or diagnosis with EPS after the first TD diagnosis. Individuals with a claim for a VMAT2 inhibitor or benztropine during the baseline period for the corresponding incident treatment cohort were also excluded for all analyses. For the objective of comparing HCRU of patients with TD who are prescribed benztropine vs VMAT2 inhibitor drugs, only individuals with no claims for a VMAT2 inhibitor during the whole study period were eligible for the benztropine-treated cohort, and individuals with no claims for benztropine during the whole study period were eligible for the VMAT2 inhibitor–treated cohort.

### Outcomes and Statistical Analysis

In part 1, individuals with TD in the benztropine-treated cohort were matched to individuals with TD in the nontreated cohort exactly using 3 confounding factors commonly used for HCRU studies available in closed claims (ie, age, sex, and the Quan adaptation of the Charlson Comorbidity Index [CCI, calculated from claims in the year prior to the first TD diagnosis date]). For each matched, nontreated individual, a pseudo-treatment index date was assigned to mimic the benztropine treatment index date. The pseudo-treatment index date was equivalent to the first TD diagnosis date plus the number of days between the first TD diagnosis date and the treatment index date for the matched individual in the benztropine-treated cohort. All-cause HCRU, including inpatient visits, emergency department (ED) visits, medications prescribed, outpatient physician visits, and outpatient nonphysician visits, was quantified and compared between cohorts using paired *t*-tests, with significance level *P* ≤ .05.

In part 2, treatment patterns including duration of persistence, adherence rates, and discontinuation rates were assessed. Adherence was defined as the proportion of days covered ≥80% in the 12-month post-index period.^38^ The Mann-Whitney test was used to generate *P* values for comparison of persistence duration between cohorts. The chi-square test was used to generate *P* values for comparison of adherence rates and discontinuation rates between cohorts.

For comparison of HCRU, individuals in the VMAT2 inhibitor–treated cohort were matched to individuals with TD in the benztropine-treated cohort using 1:1 greedy nearest-neighbor propensity score matching to minimize selection bias. Covariates of age, sex, payer type, Quan adaptation of the CCI, underlying mental health diagnosis, and time between TD diagnosis and index treatment date were balanced between the cohorts. Standardized mean differences (SMDs) between cohorts for baseline characteristics were evaluated before and after matching. HCRU was compared between cohorts for 12 months after index. The Wilcoxon signed-rank test and McNemar test were used to generate *P* values when comparing post-matched HCRU outcomes. All statistical comparisons were 2-sided, using a nominal significance level of .05. No formal adjustment for multiple comparisons was prespecified or applied. Therefore, all reported *P* values should be interpreted as nominal and exploratory rather than as controlling the family-wise Type I error rate. All statistical analyses were performed using SAS v.9.4 (Cary, North Carolina, USA).

## RESULTS

### Part 1: Benztropine vs No Treatment

Among 121 579 466 individuals with data captured in the MarketScan® commercial and Medicare claims database from July 2012 to September 2021, 6875 had ≥1 diagnosis of TD and were aged ≥18 years at first TD diagnosis (**Supplementary Figure S1**). Among 119 individuals who met all exclusion and inclusion criteria and received ≥1 benztropine treatment after the first TD diagnosis date, mean age was 50.1 years, with 16.0% ≥65 years, and benztropine-treated individuals had a mean Quan CCI of 1.10. Among 2859 individuals eligible for the nontreated cohort, mean age was 56.1 years, with 30.4% ≥65 years.

To control for confounding, each of 92 benztropine-treated individuals was matched exactly by age, sex, and Quan CCI to an individual who did not receive benztropine. Mean (SD) age of the matched benztropine-treated and nontreated cohorts was 47.7 (16.5) years; 77.2% of each cohort were female, and the mean (SD) Quan CCI was 0.60 (0.88) for both cohorts. Individuals in both the benztropine-treated cohort and the nontreated control cohort had a mean (SD) of 106.3 (217.7) days between TD diagnosis and the index treatment date.

In the 1-year period after the index treatment date, benztropine-treated individuals had twice the mean (SD) number of ED visits (1.0 [1.8]) than nontreated individuals (0.5 [1.0] visits; *P* = .0232) (**Figure 1A**). No statistically significant difference in the mean number of inpatient visits was observed. No statistically significant difference was found in the mean number of medications, excluding benztropine, prescribed to benztropine-treated individuals vs nontreated individuals (*P* = .1221) (**Figure 1B**). The most prescribed medications, calculated as the number of distinct days a medication was filled over 1 year, in the benztropine-treated cohort and the nontreated cohort are shown in **Supplementary Table S1**. The mean number of outpatient physician visits and nonphysician visits was not statistically significantly different for the benztropine-treated and nontreated cohorts (**Figure 1C**). No statistically significant differences were observed in the proportion of each cohort with inpatient, ED, or outpatient visits (**Supplementary Figure S3**).

**Figure 1.**
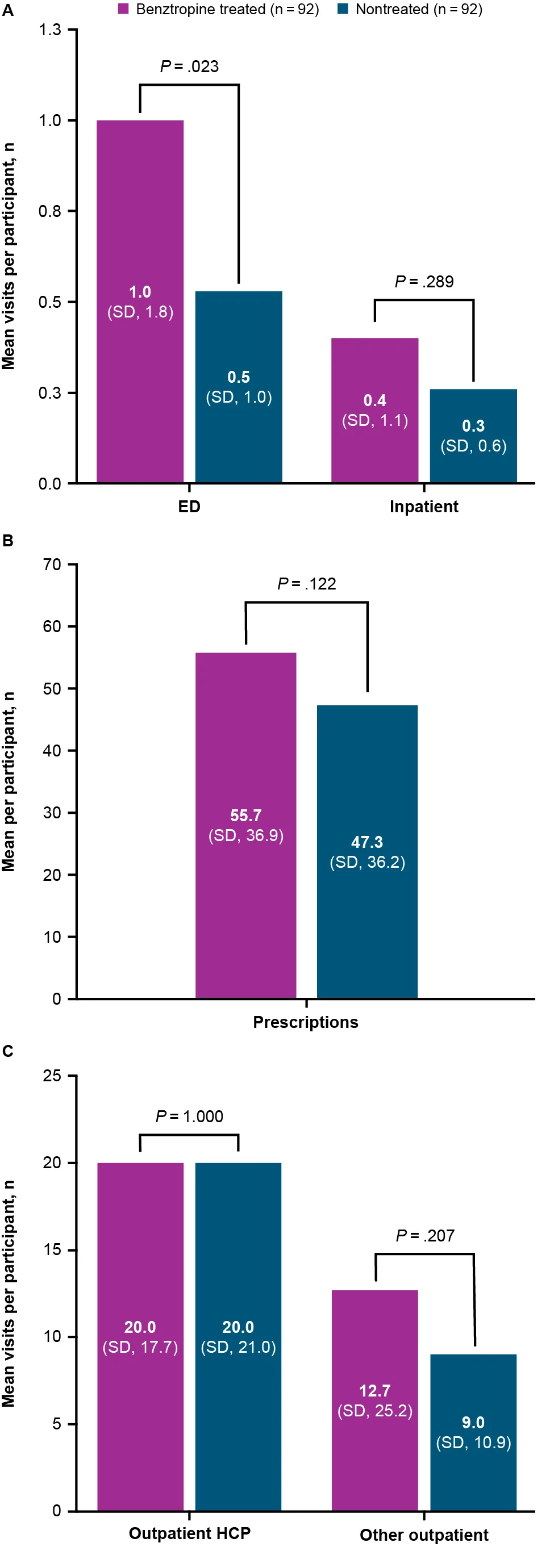
Comparison of Mean HCRU per Participant Between Matched Benztropine-Treated and Untreated Cohorts for (**A**) ED and Inpatient Visits, (**B**) Prescriptions, and (**C**) Outpatient HCP and Other Outpatient Visits Abbreviations: ED, emergency department; HCP, healthcare provider; HCRU, healthcare resource utilization.

### Part 2: Benztropine vs VMAT2 Inhibitor

In the MarketScan commercial, Medicare, and multi-state Medicaid claims databases, the number of individuals with ≥1 pharmacy claim for benztropine or a VMAT2 inhibitor during the identification period were 108 219 and 3521, respectively. Of these, 324 individuals treated with benztropine and 432 individuals treated with a VMAT2 inhibitor had 12 months of follow-up and were analyzed for treatment patterns. Mean (SD) persistence was significantly higher in those treated with a VMAT2 inhibitor (220.6 [139.7] days) than in those treated with benztropine (150.7 [142.2] days; *P* < .0001). Adherence to treatment was seen in a significantly higher proportion of individuals receiving a VMAT2 inhibitor (41.4%) compared with those receiving benztropine (24.7%; *P* < .0001). A significantly lower proportion of those receiving a VMAT2 inhibitor (58.3%) discontinued treatment compared with those receiving benztropine (73.8%; *P* < .0001).

Overall, 315 individuals in the benztropine-treated cohort and 421 individuals in the VMAT2 inhibitor–treated cohort were eligible for matching (**Supplementary Figure S2; Supplementary Table S2**), and 272 individuals in each cohort were matched 1:1 (**Table 1**). All covariates after matching had SMD <0.1, indicative of balance between the cohorts. Median age was 55.0 and 54.0 years in the matched benztropine-treated and VMAT2 inhibitor–treated cohorts, respectively. Individuals treated with benztropine were most commonly diagnosed with TD in an acute care hospital (26.1%), whereas those treated with a VMAT2 inhibitor were most commonly diagnosed with TD by a neurologist (22.4%) or nurse practitioner/psychiatric nurse (20.2%) (**Figure 2**).

**Table 1.** Baseline Characteristics of Matched Benztropine-Treated and VMAT2 Inhibitor–Treated Cohorts

**Characteristics**	**Benztropine (n = 272)**	**VMAT2 Inhibitor (n = 272)**	**SMD After Matching**
Age at index, y			0.03
Mean (SD)	51.0 (14.8)	51.4 (13.4)	
Median (range)	55.0 (18.0-98.0)	54.0 (19.0-89.0)	
Sex, n (%)			0.02
Female	182 (66.9)	185 (68.0)	
Male	90 (33.1)	87 (32.0)	
Time between TD diagnosis and index drug prescription, mean (SD), days	89.4 (118.5)	90.0 (107.9)	0.01
Payer type, n (%)			
Commercial	90 (33.1)	94 (34.6)	−0.03
Medicare	16 (5.9)	17 (6.3)	−0.01
Medicaid	166 (61.0)	161 (59.2)	0.04
Charlson Comorbidity Index (Quan version), mean (SD)	1.6 (2.0)	1.6 (2.1)	0.00
Psychotic disorders, n (%)	133 (48.9)	111 (40.8)	NA
Schizophrenia	72 (26.5)	65 (23.9)	0.06
Schizoaffective disorder	72 (26.5)	67 (24.6)	0.04
Mood disorders, n (%)	204 (75.0)	206 (75.7)	NA
Bipolar disorder	128 (47.1)	121 (44.5)	0.05
Major depressive disorder	137 (50.4)	141 (51.8)	−0.03
Unspecified mood disorder	22 (8.1)	20 (7.4)	0.03
Other mood disorders	33 (12.1)	31 (11.4)	0.02

Abbreviations: NA, not available; SMD, standardized mean difference; TD, tardive dyskinesia; VMAT2, vesicular monoamine transporter type 2.

**Figure 2.**
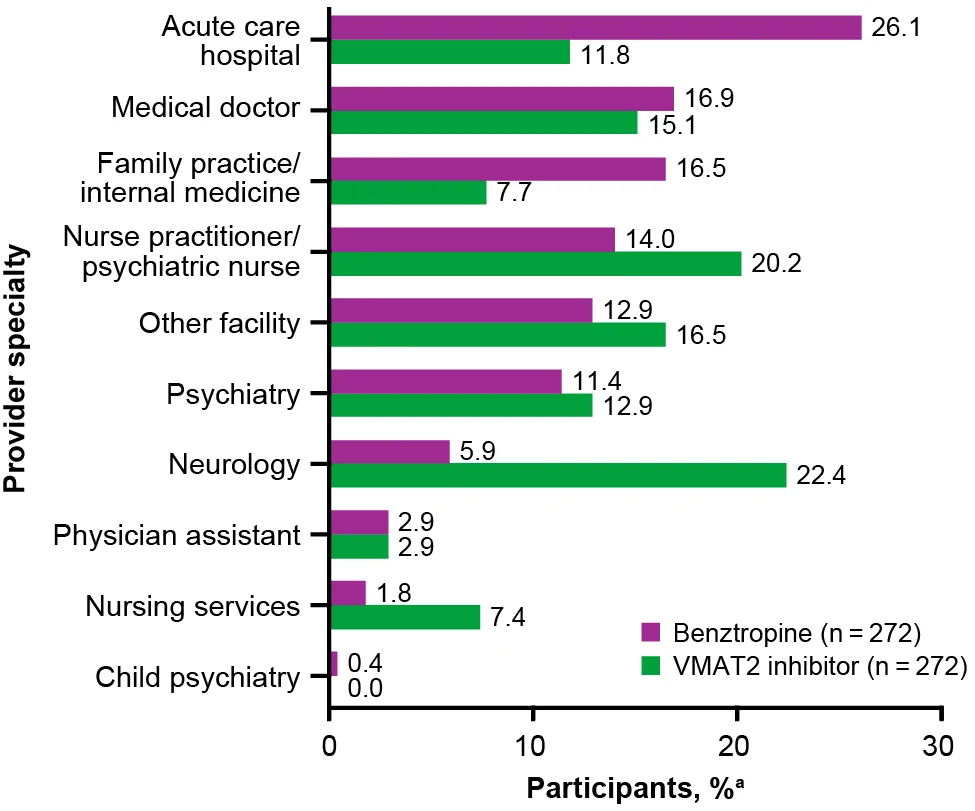
Type of Facility or Clinician at TD Diagnosis in Benztropine-Treated and VMAT2 Inhibitor–Treated Cohorts Abbreviations: TD, tardive dyskinesia; VMAT2, vesicular monoamine transporter type 2.

In the VMAT2 inhibitor–treated cohort in the 12 months after index, 60.3% of individuals had prescriptions for valbenazine only, 29.0% had prescriptions for deutetrabenazine only, and 10.7% had prescriptions for both VMAT2 inhibitors. The mean (SD) number of unique medications prescribed did not differ between the benztropine and VMAT2 inhibitor–treated cohorts (17.5 [11.6] vs 18.5 [11.4], respectively; *P* = .263). The most commonly prescribed concomitant medications within each cohort are shown in **Supplementary Table S3**.

The proportion of individuals with ≥1 inpatient visit was significantly higher in the benztropine-treated cohort compared with the VMAT2 inhibitor–treated cohort (36.8% vs 22.1%; *P* < .001) (**Figure 3A**). Individuals in the benztropine-treated cohort had a significantly higher mean (SD) number of inpatient (0.9 [2.2] vs 0.5 [1.2]; *P* = .004) and ED visits (2.6 [6.0] vs 1.6 [4.0]; *P* = .024) compared with the VMAT2 inhibitor–treated cohort (**Table 2**). A significantly higher proportion of the VMAT2 inhibitor–treated cohort had ≥1 visit to a nurse practitioner/psychiatric nurse or neurologist than in the benztropine-treated cohort. Mean (SD) number of visits to a neurologist was also significantly higher for individuals in the VMAT2 inhibitor–treated cohort compared with the benztropine-treated cohort (0.7 [1.7] vs 0.3 [1.1]; *P* < .001).

**Figure 3.**
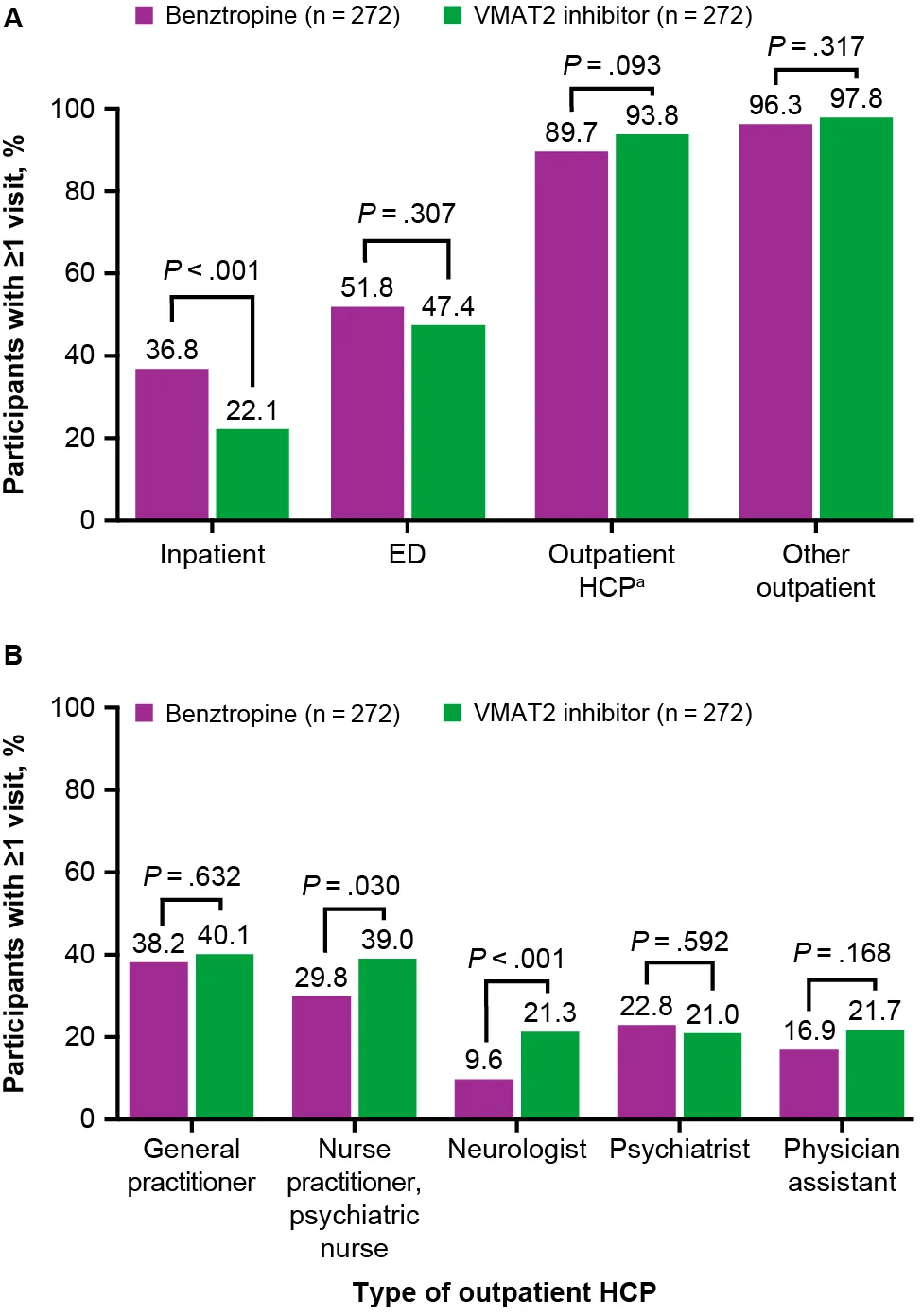
Comparison of HCRU for Matched Benztropine-Treated vs VMAT2 Inhibitor–Treated Cohorts for (**A**) Inpatient and ED Visits and Outpatient Visits and (**B**) by Type of Provider Abbreviations: ED, emergency department; HCRU, healthcare resource utilization; VMAT2, vesicular monoamine transporter type 2. ^a^Outpatient HCPs included general practitioner, nurse practitioner/psychiatric nurse, neurologist, psychiatrist, and physician assistant.

**Table 2.** Comparison of Mean HCRU Per Participant for the Benztropine-Treated and VMAT2 Inhibitor–Treated Cohorts

	**Benztropine (n = 272)**	**VMAT2 Inhibitor (n = 272)**	***P* Value**
Inpatient visits, mean (SD)	0.9 (2.2)	0.5 (1.2)	.004
ED visits, mean (SD)	2.6 (6.0)	1.6 (4.0)	.024
Outpatient HCP visits, mean (SD)	18.3 (22.6)	19.1 (17.5)	.092
Outpatient general practitioner (family practice/internal medicine)	2 (4.4)	2.1 (3.8)	.204
Outpatient nurse practitioner/psychiatric nurse	1.7 (4.5)	1.8 (3.4)	.142
Outpatient neurologist	0.3 (1.1)	0.7 (1.7)	<.0001
Outpatient psychiatrist	1.7 (5.5)	1.7 (6)	.748
Outpatient physician assistant visits	0.8 (2.4)	0.7 (1.9)	.954
Other outpatient visits, mean (SD)	44.6 (72.3)	43.4 (69.6)	.577

Abbreviations: ED, emergency department; HCP, healthcare provider; HCRU, healthcare resource utilization; VMAT2, vesicular monoamine transporter 2.

## DISCUSSION

Despite guideline recommendations and warnings in its prescribing information against using the anticholinergic benztropine for TD,^19,39^ this US claims analysis shows that benztropine has been prescribed to individuals with TD who have no other conditions that would warrant a benztropine prescription. In individuals newly diagnosed with TD in the US, benztropine prescription was associated with higher HCRU (statistically significant increases in the number of ED visits) compared with no treatment during the first year after benztropine initiation. In contrast, individuals with TD with a VMAT2 inhibitor prescription had fewer inpatient admissions and ED visits than those prescribed benztropine. Individuals treated with VMAT2 inhibitors were more likely to be diagnosed with TD by neurology specialists and nurse practitioners/psychiatric nurses than those treated with benztropine. Consistent with this, those treated with VMAT2 inhibitors had more outpatient visits to neurology specialists and nurse practitioners/psychiatric nurses compared with those treated with benztropine. The increase in outpatient visits for those receiving VMAT2 inhibitors may reflect more frequent health management of individuals receiving these medications compared with those receiving benztropine. Treatment with a VMAT2 inhibitor was also associated with higher levels of persistence and adherence than treatment with benztropine.

Benztropine was widely prescribed to individuals with TD in both parts of this claims study. Notably, individuals treated with benztropine prior to TD diagnosis and those with drug-induced parkinsonism were excluded from this claims study, suggesting that benztropine was prescribed as an intended treatment for TD. The continued widespread use of benztropine to treat TD is consistent with reports that benztropine remains the most common treatment for EPS.^40^

In this claims analysis, individuals followed by specialists (eg, neurologists and psychiatric nurse practitioners) were more frequently prescribed VMAT2 inhibitors than benztropine, yet for individuals followed by general practitioners, off-label treatments for TD were more frequently prescribed than approved VMAT2 inhibitor treatments. In an independent survey of healthcare providers who prescribe benztropine, 80% of primary care physicians and 39% of psychiatrists reported initiating benztropine to treat TD, and over one-third of all providers surveyed reported using benztropine to prevent TD,^9^ despite lack of evidence for efficacy.^18^ In a separate survey of healthcare providers, a higher proportion of psychiatric HCPs than primary care HCPs reported being familiar with the 2020 American Psychiatric Association Practice Guideline for Treatment of Patients with Schizophrenia,^41^ recommending VMAT2 inhibitors as treatment for individuals with TD.^9^ Similarly, an analysis of Medicare and Medicaid claims from 2017 to 2020 found that neurologists and psychiatrists were the primary prescribers of VMAT2 inhibitors.^42^ Taken together, these results suggest that primary care physicians may benefit from education to support differential diagnosis of DIMDs and corresponding treatment recommendations.

In prior claims studies comparing individuals with underlying psychiatric disorders with and without TD, greater HCRU was observed in those with TD.^43–45^ In several real-world studies in the US, individuals with TD have been found to have increased inpatient admissions compared with matched individuals without TD.^44–46^ In one study, increases in inpatient admissions were shown to be the main driver of increased healthcare costs in individuals with TD.^44^ Similarly, individuals with evidence of EPS claims have been shown to have increased inpatient admissions and medical costs when compared with matched individuals without EPS claims.^5,8,47^ This is the first study comparing HCRU between individuals with TD treated with benztropine matched exactly for age, sex, and comorbidities to those who were untreated. Newly diagnosed individuals with TD treated with benztropine had higher mean numbers of ED visits compared with untreated controls. Notably, those treated with benztropine had no significant improvements in HCRU when compared with matched controls. Effective management of TD symptoms would be expected to decrease the incremental increase in HCRU seen with TD. Higher HCRU in individuals treated with benztropine may be attributed to demographic and clinical variables that were not accounted for in this matching analysis. Alternatively, the higher levels of HCRU for individuals receiving benztropine may suggest that either TD is not being managed by benztropine, or adverse effects of this anticholinergic medication outweigh any therapeutic effect in patients with TD.^26,30–32^ In part 2 of this analysis, individuals treated with VMAT2 inhibitors had fewer inpatient admissions and ED visits than those treated with benztropine. These observations are consistent with previous reports that treatment with VMAT2 inhibitors is associated with fewer falls and ED visits in residents with TD in long-term care facilities.^48^ The finding that those receiving VMAT2 inhibitors had more specialist outpatient visits is expected given prior findings that neurologists and psychiatrists are among the most frequent prescribers of VMAT2 inhibitors.^42^

### Limitations

Because only those with a coded diagnosis of TD were included in these analyses, many individuals with TD may have been excluded either due to underdiagnosis or possible misdiagnosis or miscoding of TD as another DIMD or EPS. Therefore, the sample size in this study was restricted based on the limited scope of TD diagnosis and treatment, and the included population may not be representative of the general population.

Observational claims analyses can only demonstrate associations between treatment and HCRU but do not establish causation. In both parts of this study, though direct matching or propensity score matching and attrition criteria were used to minimize potential bias, other confounding variables not used in matching may have biased the results. In part 1, because very few patients met inclusion and exclusion criteria, exact matching for additional variables would have led to sample sizes too small for informative analyses. In part 2, multiple payer types/timelines (ie, MarketScan commercial and Medicare [Jan. 2016–Sept. 2023] and Medicaid [Jan. 2018–Sept. 2022]) were included to maximize the sample size for analysis. However, since cohorts after propensity matching showed minimal differences in each payer type, different claim sources are not expected to affect analysis outcomes.

Many functional variables expected to strongly influence HCRU may have resulted in meaningful differences between treatment groups but are not captured within source claims databases and thereby were not available for matching. These include measures of TD severity, psychiatric disease severity, functional status, and cognitive impairment. Additionally, any socioeconomic and employment differences between cohorts, also not captured in claims databases, are expected to influence care. Because we cannot confirm if these characteristics were balanced between the cohorts, residual confounding by these unmeasured variables cannot be excluded. Since baseline HCRU was not included in the propensity score model, there is also a possibility of residual confounding due to differences in prior HCRU.

In part 2 of this study, a higher proportion of the VMAT2 inhibitor–treated cohort were diagnosed by specialists than in the benztropine-treated cohort, and an imbalance in baseline access to care is one possible explanation for this finding. Observed differences in treatment patterns and HCRU between treatment groups should therefore be interpreted in the context of potential confounding by unmatched variables.

Several HCRU outcomes were nonnegative count variables characterized by substantial skewness, many zero values, and variances considerably larger than their means. The Wilcoxon signed-rank tests used in part 2 accounted for the matched design but did not explicitly model the mean-variance relationship of the count outcomes. In addition, the Wilcoxon signed-rank test evaluates a paired distributional difference rather than a difference or ratio in expected counts. A matched negative-binomial model could have accommodated overdispersion and provided relative mean utilization estimates. Because count-regression analyses were not performed, alternative modeling approaches could have produced different effect estimates or statistical inferences. Moreover, the study evaluated numerous correlated HCRU outcomes and provider categories without formal adjustment for multiplicity. Consequently, the nominal *P* values do not control the family-wise probability of a false-positive finding, and some statistically significant results, particularly those with *P* values close to .05, may reflect chance. The findings should therefore be considered exploratory and interpreted according to the magnitude, consistency, and clinical plausibility of the observed associations rather than statistical significance alone. Finally, as in all claims studies, caution is necessary when interpreting treatment data, as claims databases do not document unreported side effects of a medication or whether individuals actually used medication, but only that a prescription was filled.

## CONCLUSION

The results of this real-world study suggest that benztropine was associated with higher HCRU in individuals with TD compared with lack of treatment or VMAT2 inhibitor treatment. A clearer understanding of this relationship, including future studies with multiplicity-adjusted analyses, could help inform future approaches to optimize TD management for better patient outcomes.

### Disclosures

C.C. has served on the advisory boards of AbbVie, Acadia, Alkermes, Axsome, Biogen, Bristol Myers Squibb, Corium, Idorsia, Intracellular, Janssen, Karuna, Lundbeck, Moderna, Neurocrine, Noven, Otsuka, Sage, Sumitomo, and Teva; served as a consultant for AbbVie, Acadia, Alkermes, Axsome, Biogen, Bristol Myers Squibb, Corium, Intracellular, Janssen, Karuna, Lundbeck, Medin-Cell, Neurocrine, Otsuka, Sage, Sumitomo, Supernus, and Teva; has received grant or research support from Acadia, Axsome, Harmony, Neurocrine, and Teva; and has been on the speakers bureau for AbbVie, Acadia, Alkermes, Axsome, Intracellular, Janssen, Lundbeck, Merck, Neurocrine, Noven, Otsuka, and Teva. H.X. and P.G. are employees and/or shareholders of Teva Pharmaceuticals. A.Y. was an employee and/or shareholder of Teva Pharmaceuticals at the time of this study. S.C. is an employee of KMK Consulting, Inc., which has received payments from Teva Pharmaceuticals in relation to this study.

## Supporting information

Online Supplementary Material
