# Supplementary material for: Real-World Treatment Patterns and Healthcare Resource Utilization in Tardive Dyskinesia: Claims Analysis of Individuals Treated with Benztropine or a VMAT2 Inhibitor
Source: J Health Econ Outcomes Res. 2026 Nov 13;13(2):241–8. doi: 10.36469/001c.169275 (PMC13770533; doi:10.36469/001c.169275)
Supplement: Supplementary file 1 [file jheor_2026_13_2_169275_377221.pdf]

Journal of Health Economics and Outcomes Research

Online Supplementary Material

Real-World Treatment Patterns and Healthcare Resource Utilization in Tardive Dyskinesia: Claims Analysis of Individuals Treated with Benztropine or a VMAT2 Inhibitor. *JHEOR*. 2026;13(2):241-248. doi:10.36469/jheor.2026.169275

Table S1: Most Common Prescriptions Received by Benztropine-Treated and Nontreated Cohorts in the Year After Index

Table S2: Baseline Characteristics of Benztropine-Treated and VMAT2 Inhibitor–Treated Cohorts Before Matching

Table S3: Most Prescribed Concomitant Medications Received by Benztropine-Treated and VMAT2 Inhibitor–Treated Cohorts in the Year After Index

Figure S1: Study Design and Disposition Part 1: Benztropine-Treated and Nontreated Cohorts

Figure S2: Study Design and Disposition Part 2: Benztropine-Treated and VMAT2 Inhibitor–Treated Cohorts

Figure S3: Comparison of HCRU for Matched Benztropine-Treated vs Nontreated Cohorts

This supplementary material has been provided by the authors to give readers additional information about their work.

This is an open-access article distributed under the terms of the Creative Commons Attribution 4.0 International License (CCBY-4.0). View this license's legal deed at http://creativecommons.org/licenses/by/4.0 and legal code at http://creativecommons.org/licenses/by/4.0/legalcode for more information.

Table S1. Most Common Prescriptions Received by Benztropine-Treated and Nontreated Cohorts in the Year After Index

**Benztropine-Treated Cohort (n = 92) No. of Prescription Fills in 1 Year**		**Nontreated Cohort (n = 92) No. of Prescription Fills in 1 Year**	
**Medication**	**n (%)**	**Medication**	**n (%)**
Levothyroxine sodium	23 (25.0)	Acetaminophen/hydrocodone bitartrate	24 (26.1)
Gabapentin	22 (23.9)	Clonazepam	23 (25.0)
Acetaminophen/hydrocodone bitartrate	22 (23.9)	Gabapentin	19 (20.7)
Trazodone hydrochloride	22 (23.9)	Azithromycin	19 (20.7)
Clonazepam	22 (23.9)	Levothyroxine sodium	19 (20.7)

Table S2. Baseline Characteristics of Benztropine-Treated and VMAT2 Inhibitor–Treated Cohorts Before Matching

**Characteristics**	**Benztropine-Treated Cohort (n = 315)**	**VMAT2 Inhibitor–Treated Cohort (n = 421)**	**SMD Before Matching**
Age at index, y			0.31
Mean (SD)	49.5 (15.7)	54.0 (13.2)	
Median (range)	54.0 (18.0-98.0)	56.0 (19.0-89.0)	
Sex, n (%)			0.11
Female	200 (63.5)	289 (68.6)	
Male	115 (36.5)	132 (31.4)	
Time between TD diagnosis and index drug prescription, mean (SD), d	85.6 (115.6)	104.1 (117.4)	0.16
Payer type, n (%)			
Commercial	91 (28.9)	164 (39.0)	–0.21
Medicare	17 (5.4)	52 (12.4)	–0.25
Medicaid	207 (65.7)	205 (48.7)	0.35
Charlson Comorbidity Index (Quan version), mean (SD)	1.7 (2.1)	1.5 (1.9)	–0.09
Psychotic disorders, n (%)	170 (54.0)	128 (30.4)	NA
Schizophrenia	101 (32.1)	72 (17.1)	0.35
Schizoaffective disorder	98 (31.1)	73 (17.3)	0.33
Mood disorders, n (%)	240 (76.2)	329 (78.1)	NA
Bipolar disorder	147 (46.7)	201 (47.7)	–0.02
Major depressive disorder	164 (52.1)	216 (51.3)	0.02
Unspecified mood disorder	28 (8.9)	28 (6.7)	0.08
Other mood disorders	51 (16.2)	42 (10.0)	0.19

Abbreviations: NA, not applicable; SMD, standardized mean difference; TD, tardive dyskinesia; VMAT2, vesicular monoamine transporter type 2.

Table S3. Most Prescribed Concomitant Medications Received by Benztropine-Treated and VMAT2 Inhibitor–Treated Cohorts in the Year After Index

**Benztropine-Treated Cohort (n = 272)**		**VMAT2 Inhibitor–Treated Cohort (n = 272)**	
**Medication**	**n (%)**	**Medication**	**n (%)**
Trazodone hydrochloride	82 (30.1)	Trazodone hydrochloride	88 (32.4)
Atorvastatin calcium	78 (28.7)	Gabapentin	81 (29.8)
Gabapentin	71 (26.1)	Albuterol sulfate	70 (25.7)
Omeprazole	61 (22.4)	Levothyroxine sodium	60 (22.1)
Quetiapine fumarate	58 (21.3)	Atorvastatin calcium	60 (22.1)

Abbreviation: VMAT2, vesicular monoamine transporter type 2.

Figure S1. Study Design and Disposition Part 1: Benztropine-Treated and Nontreated Cohorts

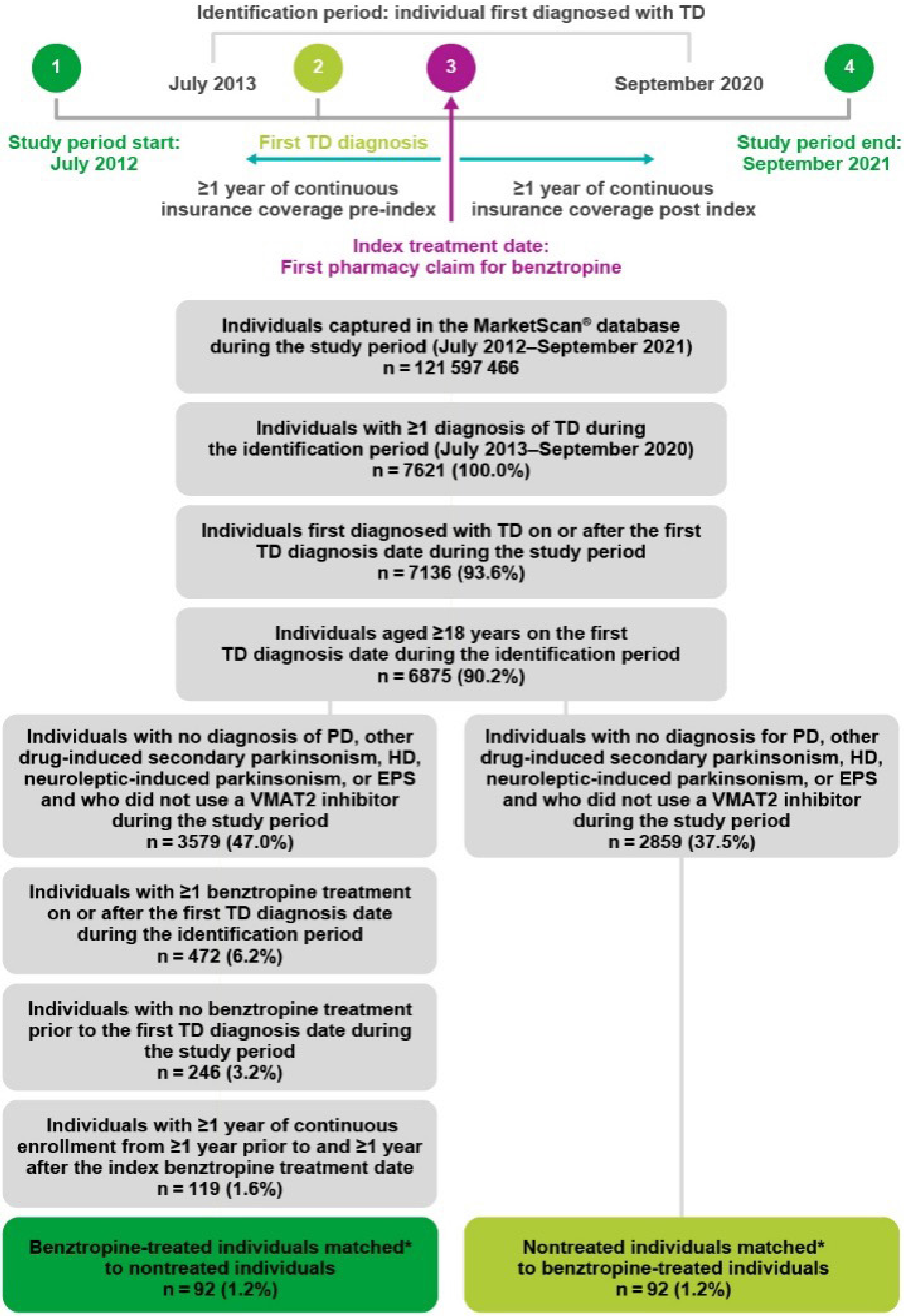

Abbreviations: EPS, extrapyramidal symptoms; HD, Huntington disease; PD, Parkinson's disease; TD, tardive dyskinesia; VMAT2, vesicular monoamine transporter type 2.

Figure S2. Study Design and Disposition Part 2: Benztropine-Treated and VMAT2 Inhibitor–Treated Cohorts

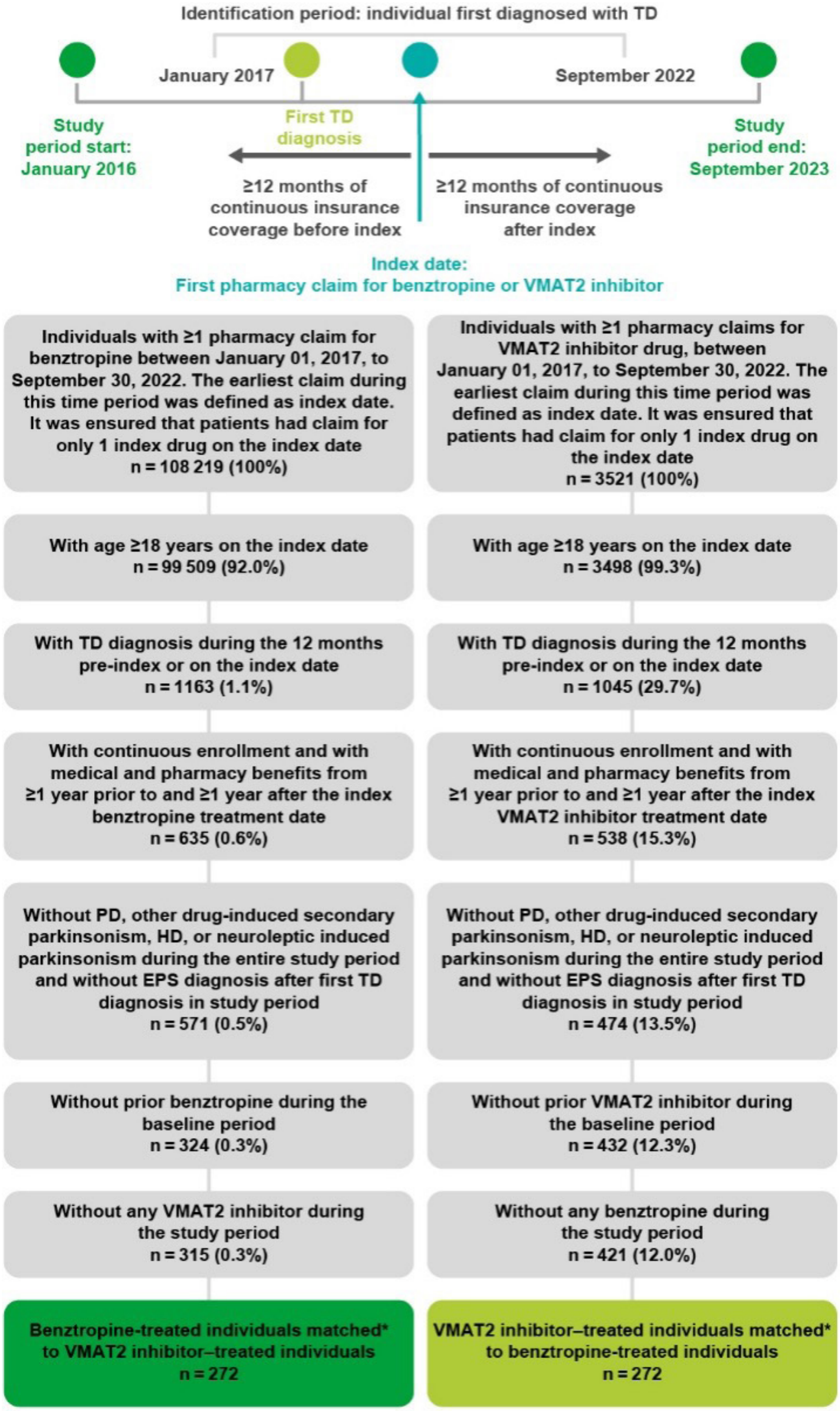

Abbreviations: EPS, extrapyramidal symptoms; HD, Huntington disease; PD, Parkinson's disease; TD, tardive dyskinesia; VMAT2i, vesicular monoamine transporter type 2.

Figure S3. Comparison of HCRU for Matched Benztropine-Treated vs Nontreated Cohorts

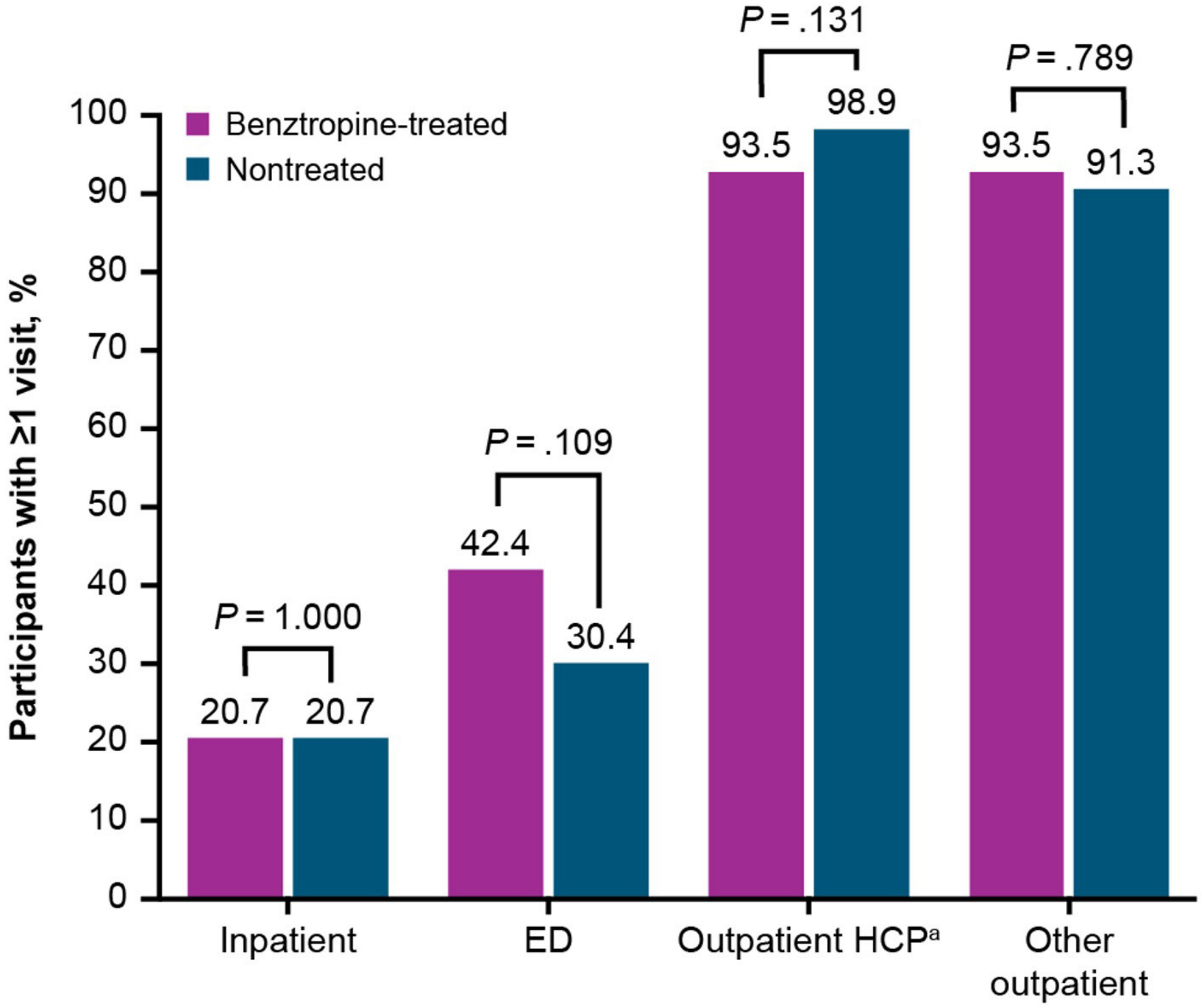

Abbreviations: ED, emergency department; HCP, healthcare provider; HCRU, healthcare resource utilization.
[a]Outpatient HCPs included general practitioner, nurse practitioner/psychiatric nurse, neurologist, psychiatrist, and physician assistant.
